# Applying the Bayesian Stackelberg Active Deception Game for Securing Infrastructure Networks

**DOI:** 10.3390/e21090909

**Published:** 2019-09-18

**Authors:** Chengyi Zeng, Baoan Ren, Hongfu Liu, Jing Chen

**Affiliations:** College of Intelligence Science and Technology, National University of Defense Technology, Changsha 410073, China

**Keywords:** infrastructure network, Bayesian Stackelberg game, asymmetry information

## Abstract

With new security threats cropping up every day, finding a real-time and smart protection strategy for critical infrastructure has become a big challenge. Game theory is suitable for solving this problem, for it provides a theoretical framework for analyzing the intelligent decisions from both attackers and defenders. However, existing methods are only based on complete information and only consider a single type of attacker, which is not always available in realistic situations. Furthermore, although infrastructure interconnection has been greatly improved, there is a lack of methods considering network characteristics. To overcome these limitations, we focus on the problem of infrastructure network protection under asymmetry information. We present a novel method to measure the performance of infrastructure from the network perspective. Moreover, we propose a false network construction method to simulate how the defender applies asymmetric information to defend against the attacker actively. Meanwhile, we consider multiple types of attackers and introduce the Bayesian Stackelberg game to build the model. Experiments in real infrastructure networks reveal that our approach can improve infrastructure protection performance. Our method gives a brand new way to approach the problem of infrastructure security defense.

## 1. Introduction

Modern society is highly dependent on infrastructure, and any failure of infrastructure will seriously affect people’s daily life. With the increase of terrorism, how to effectively prevent attacks on infrastructure has become a worthwhile subject of research. For the protection of infrastructure, researchers also provide many research methods, such as probabilistic risk assessment and historical data analysis. However, because these methods need static input in the research process, it is not suitable for the study of intelligent countermeasure behavior [1,2,3]. Game theory is a natural modeling paradigm for a multi-agent intelligent interaction scenario, which can provide an accurate individual interaction model for the research on intelligent individual confrontation. As Hall [4] mentioned, “if the conditions creating the problems you had to deal with were natural or random, the answer was decision analysis (which looked a lot like what we now call risk analysis). If the conditions creating the problems you had to deal with were the result of deliberate choice, the answer was game theory.” Feng et al. [5] proposed a game theory method to optimize the allocation of defense resources, which combines game theory with a risk assessment to optimize the allocation of limited defense resources in a city. Zhang et al. [6] analyzed the general intrusion detection system of infrastructure and proposed a game theory model for infrastructure security management. Nochenson et al. [7] obtained Nash equilibrium strategies under various cost conditions through simulation, to better provide infrastructure administrators with possible attack behavior and possible mitigation measures. Guan et al. [8] proposed a game theory model to study how the balanced allocation of the defender and the attacker depends on the budget constraints, target valuation, cost-effectiveness of investment and the basic defensive level of the target in the game. After our investigation, we found that game theory as an appropriate method of infrastructure security protection research has been paid more and more attention by researchers [6,9,10,11,12,13,14,15].

The models mentioned earlier are based on the simultaneous game model. However, the sequential game model, which is more in line with the actual situation of infrastructure protection deserves our further study. To prevent the destruction of infrastructure from affecting people’s daily life and work, the security sector always launches defense before the attack. Therefore, the sequential game model is more in line with the actual situation of infrastructure offensive and defensive confrontation. The Stackelberg game is a sequential game which closely combines theory with practice. After Vincent Conitzer and Tuomas Sandholm published the foundational paper [16] on the Stackelberg game applied in the field of security protection in 2006, a large number of applications of the Stackelberg game in various security issues will hopefully improve the intelligent decision-making solutions to complex security problems. This has been verified in practical application systems such asARMOR (Assistant for Randomized Monitoring over Routes) [17] and IRIS (Intelligent Randomization In Scheduling) [18]. In the field of security protection, when the defender’s security resources are limited and the critical protection targets cannot be completely covered by security protection, the allocation of limited security resources must be rational. In the Stackelberg game, we consider two players—the defender and the attacker. The defender, as the leader, first promises to adopt defense strategies. The follower attacker responds based on the information of defender obtained from surveillance, reconnaissance and even intelligence. The defender can gain the ’first-mover advantage’ through this sequential game, which has been proved in mathematical theory [19]. In this paper, we use the Bayesian Stackelberg game [16] to extend a single type of attacker to a variety of types of attackers and solve it via the DOBSS (Decomposed Optimal Bayesian Stackelberg Solver) algorithm [20], which is the fastest optimal algorithm for such games.

Although the Stackelberg game provides a more practical research model for security protection, there is still much room for improvement in the research on infrastructure protection. The network era has led to the emergence of network systems. Complex networks provide a suitable modeling tool for describing complex systems in human society and nature including financial networks, ecological networks, social networks and infrastructure networks [21,22,23,24]. The complexity of networks has increased exponentially. Many works have been conducted on complex networks, such as community detection [25,26], network controllability [27,28], node ranking [29,30], link prediction [31,32] and evolutionary game [33,34]. More and more infrastructures in modern society are showing their network characteristics. The functions of infrastructural networks depend to a great extent on their connectivity and topology. Some single infrastructural failures may cause destructive effects on systems. Therefore, it is necessary to model infrastructure as a network system for research. Researchers have launched research on network disintegration based on complex network theory. In 2000, Albert et al. [35] first analyzed the scale-free network disintegration problem and proposed the famous “coexistence of robustness and vulnerability” problem. After that, Holme et al. [36] summarized and compared the network disintegration effects of various disintegration strategies. In application, Quayle et al. [37] proposed a series of strategies to disturb cancer networks; Lloyd et al. [38] proposed an optimal disintegration strategy for epidemic transmission networks. However, in the real world, it is difficult to obtain complete information on a network structure, so the research on network disintegration under incomplete information has gradually attracted attention. Dezső et al. [39] proposed a biased treatment strategy against virus transmission under uncertain information. Li Jun et al. [40] studied the optimal attack problem based on incomplete information. Wu et al. [41] studied the impact of imperfect information perturbed by node degree in a certain range on network collapse. Previous research on network disintegration and protection strategies has basically been based on the success of the strategy. However, in the confrontation scenario where both the attacker and the defender exist, the strategy selection problem of the attack and defense decision makers should be solved by game theory.

Researchers have made a preliminary exploration of the application of the game model to infrastructure protection from the perspective of the network. Li et al. made a preliminary study of the attack-defense game on complex networks by using simultaneous game theory [42] and sequential game theory [43] respectively, but this problem is still worth further exploring. Our previous work has studied the attack and defense game of infrastructure networks under asymmetric information conditions [44]. In the protection of infrastructure networks, there is still a lack of research on various types of attackers.

In summary, some researchers used game theory to study infrastructure network, and some researchers began to conduct network modeling of infrastructure system. However, there is still a lack of research that combines network science and game theory to model the infrastructure system from the perspective of a network and to conduct offensive and defensive games for uncertain attacker types. When facing multiple types of attackers, how to make use of the information advantage of asymmetric information between offensive and defensive sides to improve defense payoff is the focus of this paper. We first study the infrastructure attack-defense game based on the Bayesian Stackelberg game under the condition of multiple types of attackers and asymmetric information. To our best knowledge, this idea is new.

In this paper, we evaluate the network performance from the perspective of network science. A method of constructing asymmetric information is introduced. Then, we propose an active defense approach for the defender facing multiple types of attackers based on the Bayesian Stackelberg sequential game to improve the defender’s payoffs by providing false information to the attacker.

## 2. Bayesian Stackelberg Active Deception Game Considering Multiple Types of Attackers

Consider a target network formalized in terms of an undirected graph $G\left(V,E\right)$, where *V* is the set of nodes, E⊆V×V is the set of edges. The number of nodes is $N=\left|V\right|$. $k_{i}$ is the number of adjacent edges of node $v_{i}$. Denoted by $\langle k\rangle =\frac{1}{N}\sum_{i=1}^{N}k_{i}$ the average degree of the network.

We classify players in the game as the defender and attacker. The type of defender in the model is single and the types of the attacker in the model are multiple. In the security game, as a convention the defender is usually assumed to be a female character and the attacker is assumed to be a male character.

In this section, we first introduce the motivation to research active deception defense with a Bayesian Stackelberg game in Section 2.1. Subsequently, the Stackelberg active deception game is defined in Section 2.2. Finally, we introduce the Bayesian Stackelberg active deception game considering multiple types of attackers in Section 2.3.

### 2.1. Motivation of Bayesian Stackelberg Active Deception Game

The active deception game is a game model based on asymmetric information of attack and defense, which is consistent with the fact that the target network information of attack and defense is asymmetric. Previous studies have shown that an attacker will obtain 80 percent (some say 100 percent) of the necessary information through public information or other intelligence sources before launching an attack [45]. Therefore, it is appropriate to assume that the attacker will collect complete network information before carrying out the attack. The model is studied as a sequential game model, that is to say, the defender first promises to use his mixed defense strategy, and the attacker chooses the attack strategy to maximize his payoff according to the defender’s promise. However, on the other hand, the defender does not know whether the attacker has mastered the hybrid strategy promised by the defender, which means the defender does not know whether the active deception game is a simultaneous game or a sequential game. Zhuang and Bier have proved that the leader gains ’first-mover advantage’ when the follower responds most to a single choice [19]. Therefore, the defender can choose to publish her defense strategy to force the attacker to play a sequential game. Thus in the field of security protection, a sequential game is more practical and useful than a simultaneous game.

Although we believe that the attacker has obtained enough disinformation (from the defender’s active deceptive defense) before the attack, the defender still faces the challenge brought by the uncertainty of the type of potential attacker. Therefore, to make the active deception game more realistic and credible, it is necessary to extend the Stackelberg active deception game to the Bayesian Stackelberg active deception game.

Our study is based on the Stackelberg active deception game. The basic settings of the Stackelberg active deception game are described in Section 2.2.

### 2.2. Stackelberg Active Deception Game

The Stackelberg active deception game mainly applies the asymmetric information of both sides of attack and defense. After the defender reveals the false network to the attacker, the defender first promises to use a mixed defense strategy, and then the attacker chooses the optimal strategy to obtain more attacker’s payoff. In this section, we first introduce the method for constructing false network information. Then we describe the cost model and strategies in the Stackelberg active deception game.

#### 2.2.1. Method for Constructing the False Network Information

Because the defender masters the target network, she grasps the real target network completely. However, the attacker’s mastery of network information is easily disturbed by the defender. We use α to represent the noise level of the disclosed network. Our model constructs the false network by adding $\alpha \times \left|E\right|$ false edges and reducing $\alpha \times \left|E\right|$ real edges on the true network. This construction method can keep the total number of edges in the network unchanged. Meanwhile, to control the credibility of the false network, the noise level range in this study is α∈[0,0.5]. We use $d_{i}$ to represent the displayed degree of node $v_{i}$ in the false network.

#### 2.2.2. Cost Model

Since attacking nodes can lead to more serious consequences, we assume that both attack and defense methods are targeted at nodes. An attack on a node will cause the connected edges to be removed altogether. The consumption of defending and attacking a single node is positively correlated with the degree of nodes because the degree of nodes in some infrastructure networks represents the importance of nodes to some extent. Correspondingly, the consumption of defending and attacking will increase with increasing importance. We use $c_{i}^{D}$ and $c_{i}^{A}$ to represent defense cost and attack cost of node *i*, respectively. Defense and attack costs are defined as follows:

(1) $$\begin{array}{c} c_{i}^{D}=q^{D}k_{i},c_{i}^{A}=q^{A}k_{i}\;. \end{array}$$

Among them, $q^{D}$ and $q^{A}$ are the defense cost coefficient and attack cost coefficient, respectively. Research shows that protecting a single infrastructure consumes far more resources than attacking it [45]. The defense resources allocated by the defender for node $v_{i}$ are $r_{i}^{D}=q^{D}k_{i}=c_{i}^{D}$, and the attack resources allocated by the attacker for node $v_{i}$ are $r_{i}^{A}=q^{A}d_{i}\neq c_{i}^{A}$. We define the defense resources needed to cover the whole network as

(2) $$T^{D}=\sum_{i=1}^{N}r_{i}^{D}$$

and the attack resources needed to cover the whole network as

(3) $$T^{A}=\sum_{i=1}^{N}r_{i}^{A}\;.$$

The resources available to the defender and the attacker are represented as $R^{D}$ and $R^{A}$, which are defined as follows:

(4) $$R^{D}=\theta_{D}T^{D},R^{A}=\theta_{A}T^{A}\;.$$

In our research model, the focus of our research is on the strategies adopted by both sides when the attack and defense resources are not enough to cover the whole network. Therefore, we limit the attack and defense resources by controlling the parameters $\theta_{D}$ and $\theta_{A}$ at [0,1], which is not enough to cover the whole network. When the resources allocated to the attacking and defending sides exceed the resources needed to cover the whole network, the so-called strategic game of attacking and defending loses its meaning and the maximum payoff can be obtained by directly covering all network nodes.

The aggregates of defended and attacked nodes are denoted as $V^{D}$ and $V^{A}$, respectively. The defense strategy is expressed by $D=\left[def_{1},def_{2},\cdots def_{N}\right]$, and the attack strategy is expressed by $A=\left[att_{1},att_{2},\cdots att_{N}\right]$. If the node $v_{i}\in V^{D}$, then $def_{i}=1$, otherwise $def_{i}=0$. Similarly, the representation of the attack strategy is similar to that of the defense strategy.

$C^{D}$ and $C^{A}$ represent the total cost of defense resources and attack resources, respectively. We define $C^{D}$ and $C^{D}$, and set the constraint as follows:

(5) $$C^{D}=\sum_{i=1}^{N}def_{i}r_{i}^{D}\leq R^{D}$$

and

(6) $$C^{A}=\sum_{i=1}^{N}att_{i}r_{i}^{A}\leq R^{A}\;.$$

If the node is allocated the corresponding defense resource $\left(def_{i}=1\right)$, then the node $v_{i}$ is the protected node, we assume that the protected node $v_{i}$ will never be removed. Conversely, if an unprotected node is attacked, that is, $att_{i}=1$ and $def_{i}=0$, there is a probability that the node will be removed. We define the probability of a successful attack on an unprotected node $v_{i}$ as the attack success rate:

(7) $$p_{i}^{suc}=\left\{\begin{array}{cc} 1 & d_{i}\geq k_{i}\\ \frac{d_{i}}{k_{i}} & d_{i}<k_{i} \end{array}\right.\;.$$

#### 2.2.3. Strategies

The strategy chosen by the defender and the attacker must satisfy Equations (5) and (6), respectively. The number of both players’ strategies will increase dramatically as the number of node increases. Intuitively, for any complex network, the strategy space of confronting both sides is huge. In reality, the choice of strategies for both sides can not be completely random. For the convenience of research, we shrink the space of attack strategies to a high-degree attack strategy (HAS) and a low-degree attack strategy (LAS). In HAS, according to the degree of nodes, the transition from high-degree nodes to low-degree nodes is selected in turn until defensive resources are exhausted. The purpose of HAS is to attack some high-degree nodes to achieve a better attack effect. In LAS, on the contrary, nodes are chosen from low-degree nodes to high-degree nodes. Its purpose is to attack a large number of low-cost nodes. Similarly, defense strategies are composed of two typical defense strategies, namely a high-degree defense strategy (HDS) and a low-degree defense strategy (LDS).

Denoted by vector $X=\left[x_{h},x_{l}\right]\left(x_{i}\in \left[0,1\right],x_{h}+x_{l}=1\right)$ a mixed defense strategy, where $x_{i}$ represents the probability of adopting strategy *i*, and $i\in \left\{h,l\right\}$ represents one of the two typical defense strategies—high-degree strategy or low-degree strategy. Similarly, $Y=\left[y_{h},y_{l}\right]\left(y_{i}=0\;\;or\;\;1,y_{h}+y_{l}=1\right)$ represents a pure attack strategy. Denoted by $S_{D}$ and $S_{A}$ are the defender’s mixed strategy space and the attacker’s pure strategy space, respectively.

### 2.3. Bayesian Stackelberg Active Deception Game

A Bayesian Stackelberg active deception game is a Stackelberg active deception game in which the defender faces multiple types of attackers. The defender only knows the prior probabilities of different types of attackers. In this paper, we only focus on the uncertainty of attackers’ different types, not the uncertainty of their attack performance.

In this paper, we consider only one type of defender and multiple types of attackers. Without loss of generality, we assume that the defender faces only two types of attackers. One type of attacker focuses on the overall performance of the target network and is named the ‘global-type attacker’, while the other type focuses solely on the attack success efficiency and is called the ‘local-type attacker’.

Define *T* as the set of possible types of attackers and define *P* as the prior probability of different types of attackers. In our study, $T=\left\{g(global),l(local)\right\}$, correspondingly, $P=\left[p^{g},p^{l}\right]=\left[p^{g},1-p^{g}\right]$. For a given attacker type t∈T, suppose $D^{t}\left(X,Y\right)$ be the defender’s payoff function when the defender chooses strategy *X*, and the t-type attacker adopts pure strategy *Y*.

The measure function of network performance is denoted by Γ. In this paper, we adopt the size of the largest connected component as the measure function.

Thus, the defender’s payoff function is

(8) $$D\left(X,Y\right)=\frac{\Gamma \left(\hat{G}\right)}{\Gamma \left(G\right)}\in \left[0,1\right]\;.$$

The defender pays attention to the defense effect on the target network—the smaller $\Gamma \left(\hat{G}\right)$ is, the smaller the payoff. Among them, $\hat{G}$ denotes the size of the largest connected component of the network remained after the confrontation.

The global-type attacker focuses on network performance, so we define the payoff function of the attacker as

(9) $$A^{g}\left(X,Y\right)=\frac{\Gamma \left(G\right)-\Gamma \left(\hat{G}\right)}{\Gamma \left(G\right)}\in \left[0,1\right]\;.$$

The local-type attacker focuses on the attack success efficiency, so we define the payoff function of the attacker as

(10) $$A^{l}\left(X,Y\right)=\frac{\sum_{i\in V_{remove}}k_{i}}{R^{A}}\in \left[0,1\right]\;.$$

**Figure 1:**
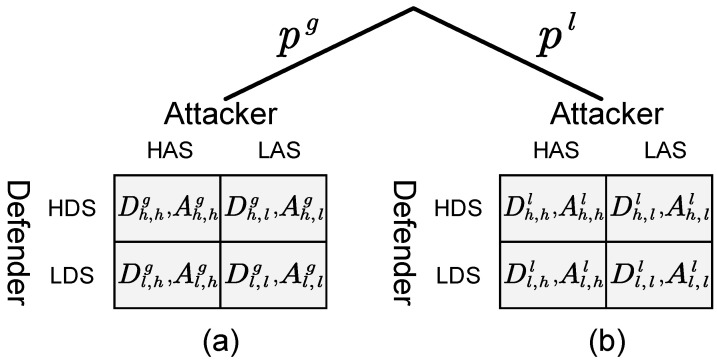
Payoff matrix of Bayesian Stackelberg active deception game. (**a**) Payoff matrices of the defender and the global-type attacker; (**b**) Payoff matrices of the defender and the local-type attacker.

Among them, $V_{remove}$ is a set of nodes removed after a successful attack from the attacker’s perspective. According to the above method, we can obtain the payoff matrix of the defender and the two types of attackers under all strategy interactions $\left|S_{A}\times S_{D}\right|$ which is shown in Figure 1. In Figure 1a, $D_{i,j}^{g}$ is the payoff of the defender when facing the global-type attacker—the defender chooses strategy *i* and the attacker adopts strategy *j*. At this time, the payoff of the attacker is $A_{i,j}^{g}$. The row and column players are the defender and attacker, respectively. Similarly, when the defender faces the local-type attacker, the payoff matrix of the defender and the attacker are $D_{i,j}^{l}$ and $A_{i,j}^{l}$, respectively, as shown in Figure 1b.

A Bayesian Stackelberg Equilibrium $\left(X^{*},Y^{1},Y^{2},\cdots ,Y^{\left|T\right|}\right)$ for the Bayesian Stackelberg active deception game is defined by Equations (11) and (12).

(11) $$\begin{array}{c} X^{*}=arg\max_{X\in S_{D}}\sum_{t\in T}p^{t}D^{t}\left(X,Y^{t}\left(x\right)\right) \end{array}$$

(12) $$\begin{array}{c} Y^{t}\left(X\right)=arg\max_{Y^{t}\in S_{A}}A^{t}\left(X,Y^{t}\right) \end{array}$$

## 3. Solving the Active Deception Game Considering Multiple Types of Attackers

In game theory, the most common solution concept is Nash equilibrium. Under this equilibrium strategy combination, any participant cannot increase his own payoff by unilaterally changing the strategy [46]. Stackelberg equilibrium is a refinement of the Nash equilibrium concept in a Stackelberg game. In this equilibrium, each player will choose the best response in each sub-game of the original game. But when multiple strategies are not different for followers, the concept cannot guarantee a unique solution. In order to obtain the unique solution, Leitmann [47] proposed two concepts of Stackelberg equilibrium, which were named “Strong Stackelberg equilibrium” (SSE) and “Weak Stackelberg equilibrium” (WSE). Strong Stackerlberg equilibrium exists in all Stackelberg games, while Weak Stackerlberg equilibrium does not necessarily exist [48].

In the case that multiple types of attackers are considered, the attacker chooses the optimal attack strategy after knowing the defense plan of the defender, and then the Bayesian active deception game can be solved by Bayesian Stackelberg equilibrium (BSE).

After obtaining the payoff matrices of the defender and two types of attackers, we introduce an efficient exact method known as DOBSS (Decomposed Optimal Bayesian Stackelberg Solver) to calculate the Bayesian Stackelberg game equilibrium (BSE) [20].

The defender’s strategy we denote by *X*, which consists of a vector of probability distributions over the defender’s pure strategies. Hence, the value $x_{i}$ is the proportion of times in which pure strategy *i* is used. Denote by $Y^{t}$ the vector of strategies of attacker type t∈T. We also denote the payoff of the defender and each of the attacker types *t* by $D_{ij}^{t}$ and $A_{ij}^{t}$. Let *M* be a large positive number. Assume that the prior probability of the *t*-type attacker the defender is facing is $p^{t}$, with $t\in \left\{g(global),l(local)\right\}$, the defender solves the following:

(13) $$\begin{array}{cc} \max_{x,y,a} & \sum_{i\in S_{D}}\sum_{t\in T}\sum_{j\in S_{A}}p^{t}D_{ij}^{t}x_{i}y_{j}^{t}\\ s.t. & \sum_{i\in S_{D}}x_{i}=1\\ & \sum_{j\in S_{A}}y_{j}^{t}=1\\ & 0\leq \left(m^{t}-\sum_{i\in S_{D}}A_{ij}^{t}x_{i}\right)\leq \left(1-y_{j}^{t}\right)M\\ & x_{i}\in \left[0,1\right]\\ & y_{j}^{t}\in \left\{0,1\right\}\\ & m^{t}\in ℜ\;. \end{array}$$

Among them, the prior probability of the occurrence of t-type attackers is represented by $p^{t}$. $x_{i}$ represents the probability that the defender adopts strategy *i* in the mixed defense strategy. $y_{j}^{t}$ represents the probability that the t-type attacker adopts strategy *j* in a pure attack strategy. Constraints 1 and 4 jointly restrict the value range of the probability of adopting various defense strategies in the mixed strategy of the defender to be [0,1], and the sum of the probabilities of various defense strategies to be 1. Constraints 2 and 5 indicate that the attacker chooses the attack strategy after mastering the defense strategy of the defender, so the pure attack strategy is adopted. The t-type attacker chooses only one strategy as the optimal response strategy in the strategy set $S_{A}$ and $y_{j}^{t}$ can only be 0 or 1. In constraint 3, since $y_{j}^{t}$ is 0 or 1, when $y_{j}^{t}$ is 0, since *M* is a large positive number, the right part of the constraint is always satisfied, and the left part of the constraint requires $m^{t}\geq \sum_{i\in S_{D}}A_{ij}^{t}x_{i}$. When $y_{j}^{t}$ is 1, constraint 3 requires $m^{t}=\sum_{i\in S_{D}}A_{ij}^{t}x_{i}$. In other words, given the mixed defensive strategy *x* of the defender, $m^{t}$ is the upper bound of the t-type attacker’s payoff.

We can linearize the quadratic programming problem Equation (13) through the change of variables $z_{ij}^{t}=x_{i}y_{j}^{t}$, thus obtaining the following mixed-integer linear programming problem:

(14) $$\begin{array}{cc} \max_{y,z,a} & \sum_{i\in S_{D}}\sum_{t\in T}\sum_{j\in S_{A}}p^{t}D_{ij}^{t}z_{ij}^{t}\\ s.t. & \sum_{i\in S_{D}}\sum_{j\in S_{A}}z_{ij}^{t}=1\\ & \sum_{j\in S_{A}}z_{ij}^{t}\leq 1\\ & y_{j}^{t}\leq \sum_{i\in S_{D}}z_{ij}^{t}\leq 1\\ & \sum_{j\in S_{A}}y_{j}^{t}=1\\ & 0\leq \left(m^{t}-\sum_{i\in S_{D}}A_{ij}^{t}\left(\sum_{h\in S_{A}}z_{ih}^{t}\right)\right)\leq \left(1-y_{j}^{t}\right)M\\ & \sum_{j\in S_{A}}z_{ij}^{t}=\sum_{j\in S_{A}}z_{ij}^{1}\\ & z_{ij}^{t}\in \left[0,1\right]\\ & y_{j}^{t}\in \left\{0,1\right\}\\ & m^{t}\in ℜ\;. \end{array}$$

From the above BSE solving process, it can be seen that the BSE calculated by the DOBSS algorithm is also a Strong Stackelberg Equilibrium. It’s just that the number of followers goes from one to multiple with a prior probability.

## 4. Experiments in Scale-Free Network

### 4.1. Game Equilibrium of Active Deception Defense Game

Because power-law networks exist widely in natural networks, a large number of infrastructure networks show power-law characteristics. Therefore, our research takes the scale-free network as the research object. The experimental object in this paper is the scale-free network ($N=500,\langle k\rangle =6$) constructed by the BA model [49]. Our experimental results are obtained from the average of 1000 independent repeated experiments.

**Figure 2:**
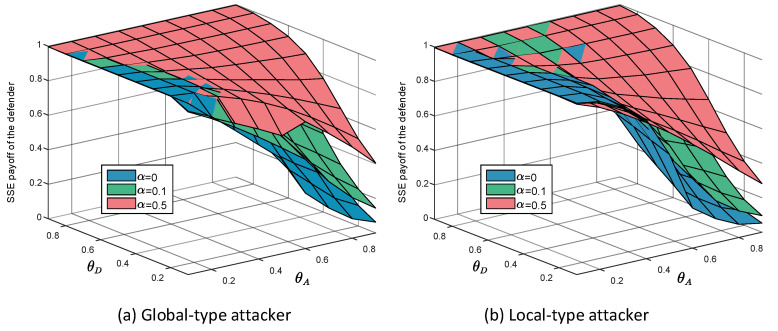
Stackelberg equilibrium payoffs of the defender under different α when the defender faces the two types of attackers separately. The target network is a scale-free network whose N=500 and $\langle k\rangle =6$.

Figure 2 shows the equilibrium payoffs when the noise level α equals 0,0.1,0.5, and the defender faces the global-type attacker and the local-type attacker separately. It can be seen that when the level of noise increases continuously, the equilibrium payoffs of the defender facing two different types of attackers also increase.

**Figure 3:**
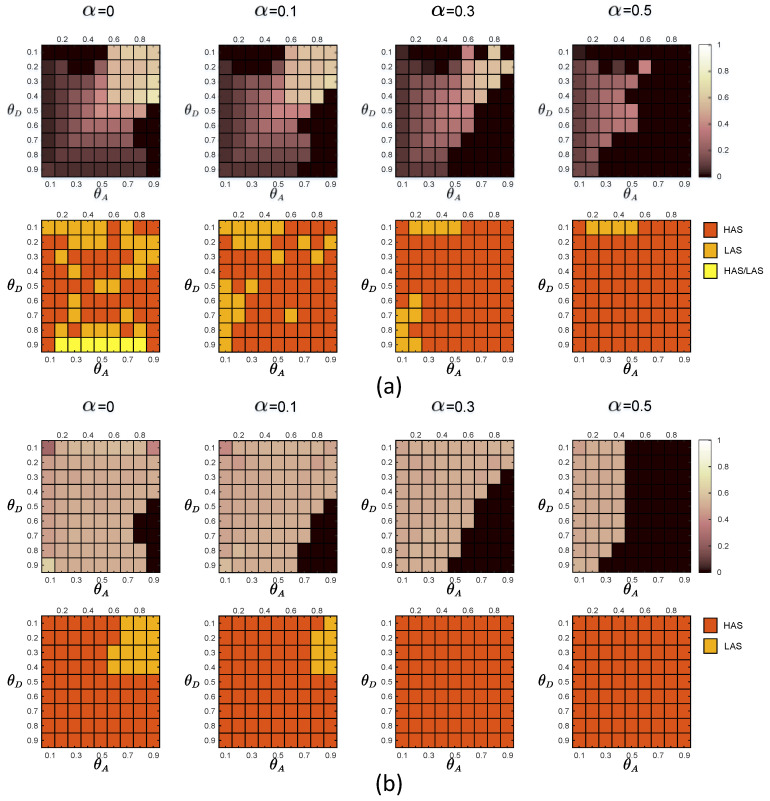
Equilibrium strategies of the defender and the attacker when there are two types of attackers. In the experiments, the defense budget constraint coefficient $\theta_{D}\in \left[0.1,0.9\right]$, the attack budget constraint coefficient $\theta_{A}\in \left[0.1,0.9\right]$. The first line in each subgraph is a graphical representation of the defender’s equilibrium defense strategy. The color of the square represents the probability of adopting high-degree defense strategy (HDS) in the mixed defense strategy. The second line in each subgraph is an illustration of the attacker’s equilibrium attack strategy. The red squares represent high-degree attack strategy (HAS), the orange squares represent low-degree attack strategy (LAS), and the yellow squares represent the same equilibrium payoff of the defender regardless of whether the attacker plays HAS or LAS. (**a**) Equilibrium strategies of the defender and the attacker when the defender faces the global-type; (**b**) Equilibrium strategies of the defender and the attacker when the defender faces the local-type attacker.

In Figure 3, we show the sequential game equilibrium strategy of the defender and the attacker when the noise level α equals 0,0.1,0.3 and 0.5. Figure 3a,b are equilibrium strategies for the defender and the attacker when the defender faces the global-type and local-type attacker separately. The first line in each subgraph is a graphical representation of the defender’s equilibrium defense strategy. The color of the square represents the probability of adopting HDS in the mixed defense strategy. The lighter the color of the square, the higher the probability of HDS in the equilibrium defense strategy. The darker the color, the lower the probability of representing LDS in an equilibrium defense strategy. The second line in each subgraph is an illustration of the attacker’s equilibrium attack strategy. The red squares represent HAS, the orange squares represent LAS, and the yellow squares represent the same equilibrium payoff of the defender regardless of whether the attacker plays HAS or LAS.

We can find that, with the increase of noise level α, the attack strategies of the two types of attackers gradually tend to choose HAS. We obtain that the displayed degree expectation of the node $v_{i}$ is

(15) E(di)=ki−E(kicut)+E(kiadd)=ki−kiα+N−1−kiαEN2−E=ki+αN−1E−N2kiN2−E=1−αN−1N2−EN2ki+αN−1EN2−E.

**Figure 4:**
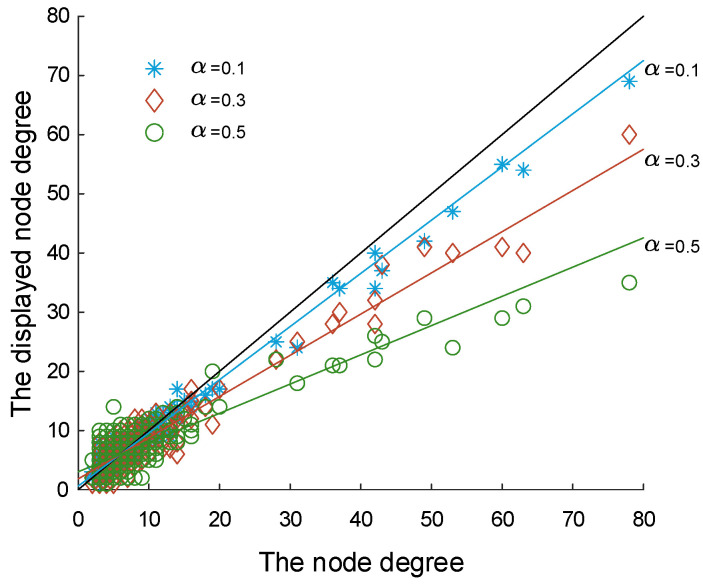
Degree pairs of nodes before and after construction of false network when α=0.1,0.3 and 0.5. The black line is the reference line which represents that $d_{i}=k_{i}$.

Among them, $E(k_{i}^{cut})$ is the expected degree deduction of node $v_{i}$ and $E(k_{i}^{add})$ is the expected increased degree of node $v_{i}$. We randomly select the false network generated at different noise levels. As shown in Figure 4, we can see that the change trend of nodes’ displayed degree matches the conclusions we deduced.

From Figure 4, we observed that nodes with a high degree have different degrees of decline in different noise levels, which is matched with Equation (4). Combined with Equation (7), we find that the false network used in our model does not change the ranking of node degree in the network, but only changes the displayed node degree $d_{i}$ [44].

### 4.2. Game Equilibrium of Bayesian Active Deception Defense Game

**Figure 5:**
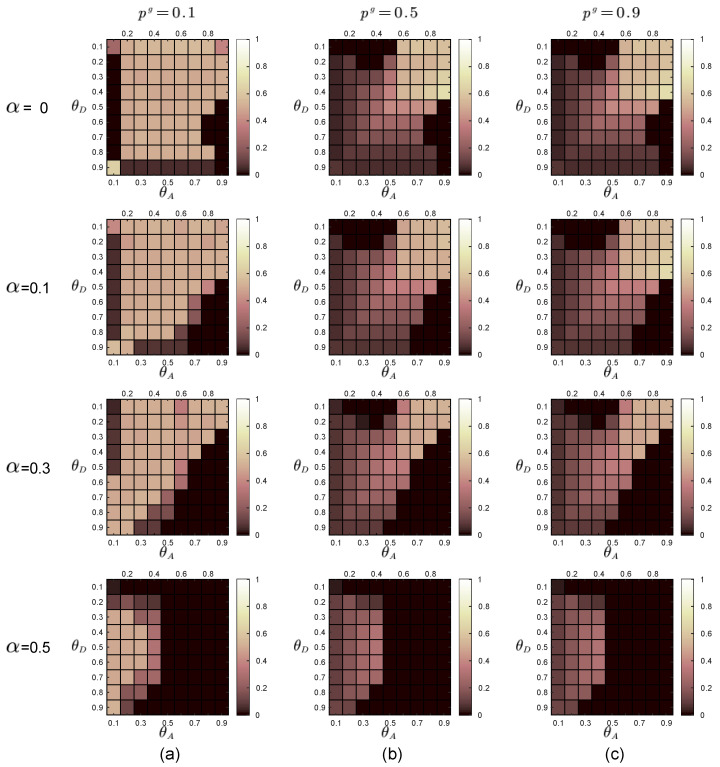
Equilibrium strategies of the defender when the defense budget constraint coefficient $\theta_{D}\in \left[0.1,0.9\right]$, the attack budget constraint coefficient $\theta_{A}\in \left[0.1,0.9\right]$. The numbers represented by colors in the blocks are the probabilities of the HDS in the defender’s mixed-Strong Stackelberg Equilibriums (SSEs). (**a**) P=[0.1,0.9]. (**b**) P=[0.5,0.5]. (**c**) P=[0.9,0.1].

**Figure 6:**
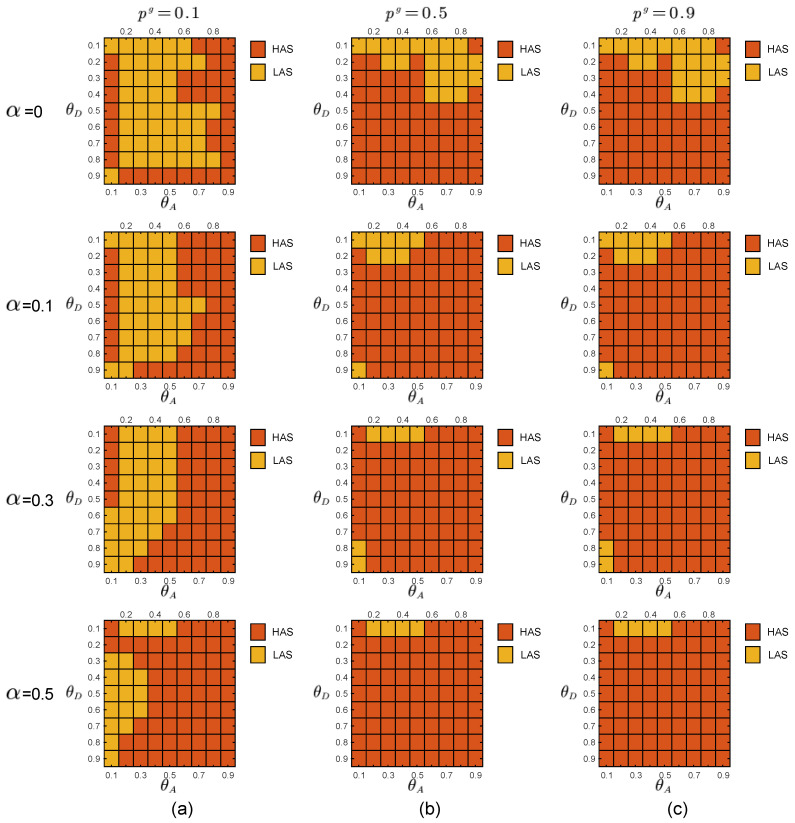
Equilibrium strategies of the global-type attacker when the defense budget constraint coefficient $\theta_{D}\in \left[0.1,0.9\right]$, the attack budget constraint coefficient $\theta_{A}\in \left[0.1,0.9\right]$. The red and orange blocks represent HAS and LAS, respectively. (**a**) P=[0.1,0.9]. (**b**) P=[0.5,0.5]. (**c**) P=[0.9,0.1].

**Figure 7:**
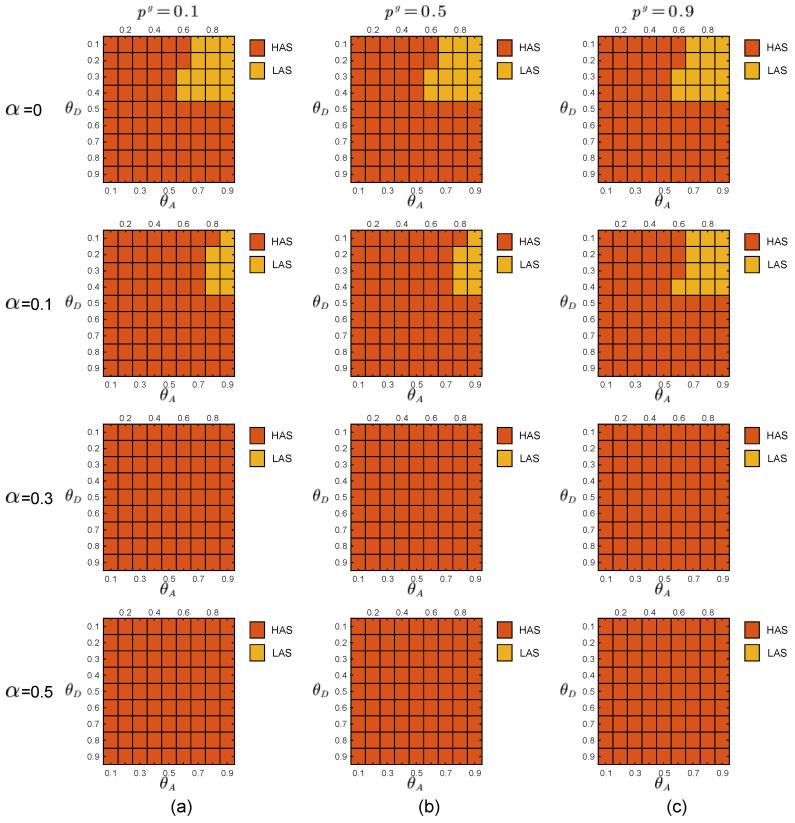
Equilibrium strategies of the local-type attacker when the defense budget constraint coefficient $\theta_{D}\in \left[0.1,0.9\right]$, the attack budget constraint coefficient $\theta_{A}\in \left[0.1,0.9\right]$. The red and orange blocks represent HAS and LAS, respectively. (**a**) P=[0.1,0.9]. (**b**) P=[0.5,0.5]. (**c**) P=[0.9,0.1].

In the experiments, the defender uses the prior probabilities of the two attacker types to calculate the payoff matrices of the defender and the two types of attackers through the function in Section 2.3. By adopting the DOBSS, we get the defender’s mixed defense strategy, the global-type attacker’s pure attack strategy and the local-type attacker’s pure strategy, as shown in Figure 5, Figure 6 and Figure 7, respectively.

We observe that when $p^{g}=p^{l}=0.5$, the defender’s defense equilibrium strategies are basically the same as the defender’s defense equilibrium strategies when facing only the global type of attacker. From the side, it can be seen that the global-type attacker poses a more significant threat to the defender, which is consistent with the global-type attacker’s efforts to reduce network performance.

On the other hand, when the noise level α is increasing, the attacker tends to adopt the HAS strategy. With the increasing noise level, the defender can use the ‘first-mover advantage’ to induce the attacker to adopt HAS with reduced attack success rate, to achieve the goal of improving the equilibrium payoff of the defender.

Taking $p^{g}=0.1$ and 0.9 as examples, we show the experimental results at $\theta_{D}=0.3$ and $\theta_{A}=0.8$. When $p^{g}=0.1$ and $p^{l}=0.9$, the probability of occurrence of a local-type attacker is large, and the probability of a global-type attacker is small. When the noise level α equals 0,0.1,0.3 and 0.5, respectively, the defender equilibrium mixed strategy $\left(x_{h},x_{l}\right)$ is (0.4995,0.5005),(0.502,0.498),(0.5016,0.4984) and (0,1), respectively. Observing the defender’s committed mixed strategy, if the attacker is the global-type, his best response would be playing HAS, HAS, HAS and HAS when the noise level α=0,0.1,0.3 and 0.5, respectively. While if the attacker is the local-type, his best response would be playing LAS, LAS, HAS and HAS when the noise level α=0,0.1,0.3 and 0.5, respectively. We can obtain the payoffs of the players on the Bayesian Stackelberg equilibrium. If the attacker is the global-type, the defender’s payoff would be 0.2607,0.385,0.5708 and 0.6999, the global-type attacker’s payoff would be 0.8106,0.7898,0.7655 and 0.9529; if the attacker is the local-type, the defender’s payoff would be 0.2434,0.3098,0.4311 and 0.5632, while the local-type attacker’s payoff would be 0.7501,0.7504,0.7506 and 0.8737 when the noise level α=0,0.1,0.3 and 0.5, respectively. Therefore, by adopting the mixed defense strategy resulting from the Bayesian Stackelberg equilibrium, the defender’s expected payoff is 0.2451, 0.3173, 0.4451 and 0.5769 when the noise level α=0,0.1,0.3 and 0.5, respectively.

When $p^{g}=0.9$ and $p^{l}=0.1$, the probability of occurrence of a global-type attacker is large, and the probability of a local-type attacker is small. When the noise level α equals 0,0.1,0.3 and 0.5, respectively, the defender equilibrium mixed strategy $\left(x_{h},x_{l}\right)$ is (0.5762,0.4238),(0.5728,0.4272),(0.5016,0.4984) and (0,1), respectively. Observing the defender’s committed mixed strategy, if the attacker is the global-type, his best response would be playing LAS, HAS, HAS and HAS when the noise level α=0,0.1,0.3 and 0.5, respectively. While if the attacker is the local-type, his best response would be playing LAS, LAS, HAS and HAS when the noise level α=0,0.1,0.3 and 0.5, respectively. We can obtain the payoffs of the players on the Bayesian Stackelberg equilibrium. If the attacker is the global-type, the defender’s payoff would be 0.301,0.4114,0.5708 and 0.6999, the global-type attacker’s payoff would be 0.7829,0.7623,0.7655 and 0.9529; if the attacker is the local-type, the defender’s payoff would be 0.2169,0.283,0.4311 and 0.5632, while the local-type attacker’s payoff would be 0.7693,0.7684,0.7506 and 0.8737 when the noise level α=0,0.1,0.3 and 0.5, respectively. Therefore, by adopting the mixed defense strategy resulting from the Bayesian Stackelberg equilibrium, the defender’s expected payoff is 0.2926,0.3986,0.5568 and 0.6862 when the noise level α=0,0.1,0.3 and 0.5, respectively.

### 4.3. Sensitiveness Analysis

**Figure 8:**
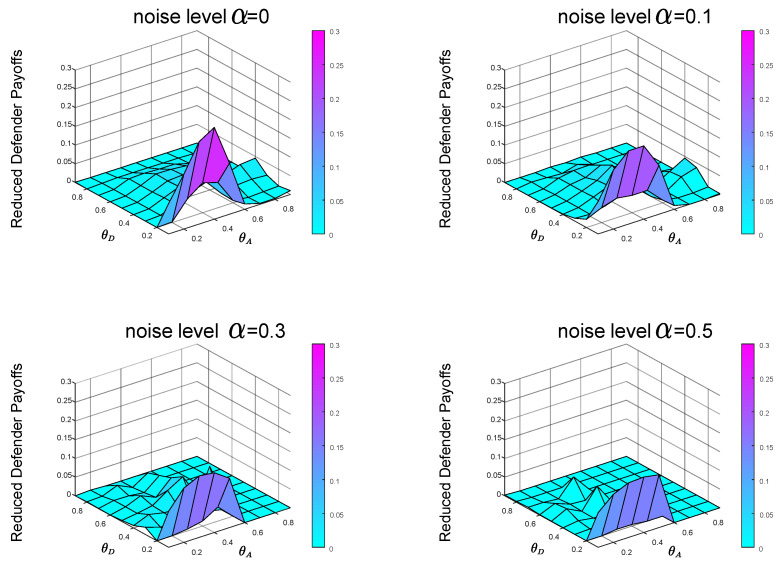
$P^{\prime }=\left[0.5,0.5\right]$, P=[0.1,0.9]. The data on the Z-axis in the figure represents the decrease in the defender’s payoffs in the case of prior probability’s misjudgment compared with that in the case of accurate prior probability *P* as input.

**Figure 9:**
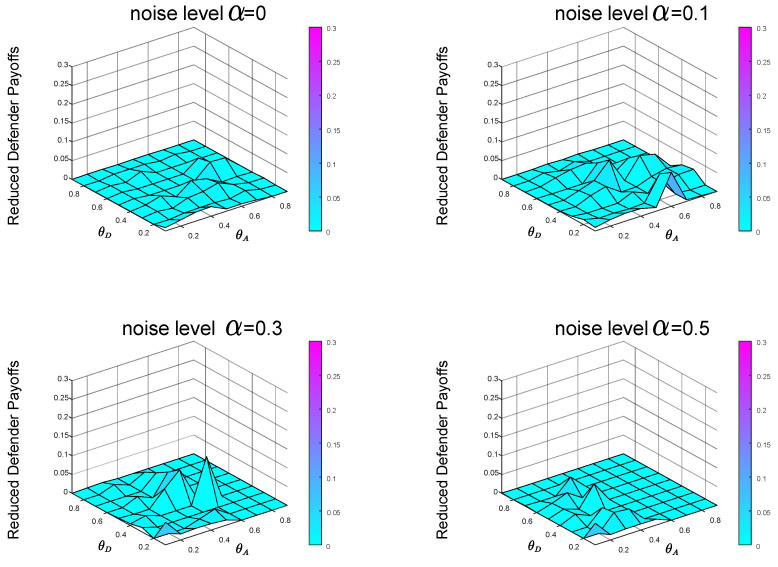
$P^{\prime }=\left[0.5,0.5\right]$, P=[0.9,0.1]. The data on the Z-axis in the figure represents the decrease in the defender’s payoffs in the case of prior probability’s misjudgment compared with that in the case of accurate prior probability *P* as input.

From the previous experiments and analysis, we know that before calculating the Bayesian Stackelberg game equilibrium, the defender must obtain the prior probability of multiple types of attackers. However, in real life, it is difficult for the defender to estimate the exact prior probability $P=[p_{g}^{exact},1-p_{g}^{exact}]$. The defender must infer the occurrence probability of multiple types of attackers through data. We define the defender’s estimate of *P* as $P^{\prime }=\left[p_{g}^{estimate},1-p_{g}^{estimate}\right]$. Then, the defender employs the Bayesian Stackelberg active defense game and uses $P^{\prime }$ as input to calculate her optimal strategy. We calculate the equilibrium defense strategy that the defender considers to be optimal at this time and then calculate the real expected defender payoff by the accurate prior probability *P*. We compare the calculated real expected defender payoff with the defender’s equilibrium payoff, calculated with the accurate prior probability *P* as input. To test the sensitivity of the equilibrium payoff to prior probability *P*, we present two groups of experimental data. In the first group of experiments, $p_{g}^{estimate}=0.5$, $p_{g}^{exact}=0.1$. The experimental result is shown in Figure 8. In the other group, $p_{g}^{estimate}=0.5$, $p_{g}^{exact}=0.9$. The experimental result is shown in Figure 9.

In the first group of experiments, in the case of $P^{\prime }=\left[0.5,0.5\right]$, P=[0.1,0.9], when the noise levels are 0,0.1,0.3 and 0.5, respectively, the average payoffs of reduction are 0.0230,0.0211,0.0162 and 0.0103, respectively. In the other group of experiments, in the case of $P^{\prime }=\left[0.5,0.5\right]$, P=[0.9,0.1], when the noise levels are 0,0.1,0.3 and 0.5, respectively, the average payoffs of reduction are 0.0054,0.0102,0.0072 and 0.0050, respectively.

Comparing the defender payoff reductions in both cases, we find that the defender payoff reductions in the first group of experimental data are significantly greater than the second group of experimental data. We know that in the first case, the defender’s defender strategy is the same as in Figure 5b. The actual optimal mixed defense strategy is consistent with the Figure 5a. In the second case, the defender’s defender strategy is still the same as the Figure 5b, and the optimal mixed strategy in the actual situation is consistent with the Figure 5c. The optimal pure attack strategy for both types of attackers is the same in both cases, consistent with the Figure 6b and the Figure 7b.

From the defender’s strategy, the defense strategy adopted by the defender after misjudging the prior probability of the attacker type is closer to the overall trend of the optimal defense strategy in the second case.

**Figure 10:**
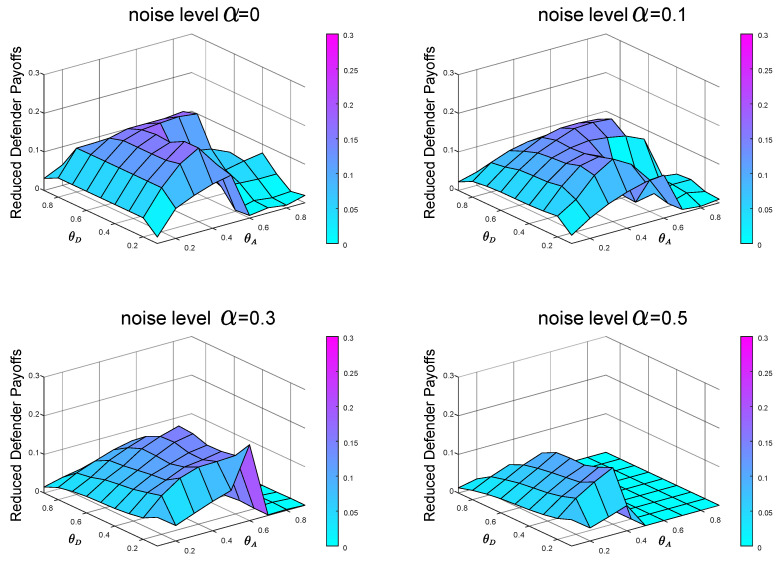
The defender misjudges that the attacker is the local type, but is actually the global type. The data on the Z-axis in the figure represents the decrease of the defender’s payoffs in the case of misjudgment compared with that in the case of an accurate judgment of the attacker’s type. $P^{\prime }=\left[0,1\right]$ and P=[1,0].

**Figure 11:**
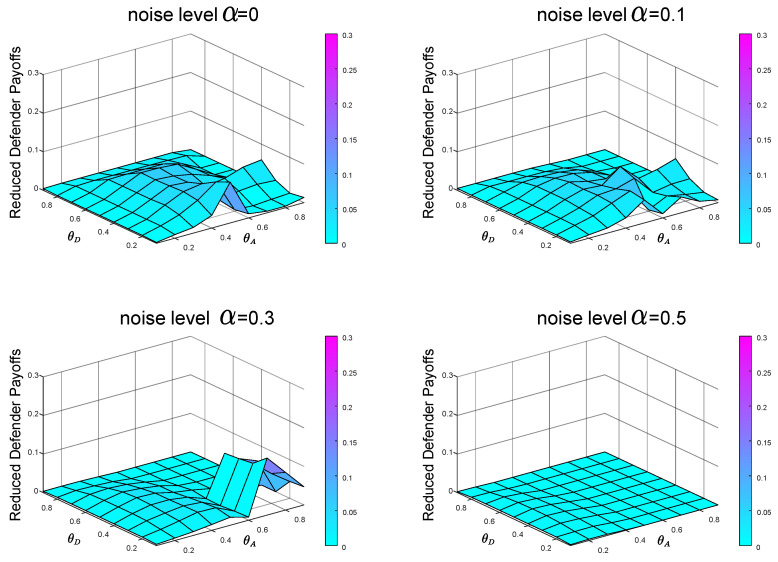
The defender misjudges that the attacker is the global type, but is actually the local type. The data on the Z-axis in the figure represents the decrease of the defender’s payoffs in the case of misjudgment compared with that in the case of an accurate judgment of the attacker’s type. $P^{\prime }=\left[1,0\right]$ and P=[0,1].

In the most extreme case, the defender does not take into account two types of attackers. The defender’s misjudgment of the attacker’s type inevitably leads to the decline of defender’s payoff. We conducted experiments on the situation of complete misjudgment. In Figure 10, the defender misjudges that the attacker is the local type, but is actually the global type, that is, $P^{\prime }=\left[0,1\right]$ and P=[1,0]. In Figure 11, the defender misjudges that the attacker is the global type, but is actually the local type, that is, $P^{\prime }=\left[1,0\right]$ and P=[0,1]. From Figure 10 and Figure 11, we can see that misjudgment of the attacker type will bring about a decrease in the defender’s payoff. Therefore, it is necessary to study the situation of the defender facing multiple types of attackers.

In the case of $P^{\prime }=\left[0,1\right]$, P=[1,0], when the noise levels are 0,0.1,0.3 and 0.5, respectively, the average payoffs of reduction are 0.0854,0.0719,0.0592 and 0.0296, respectively. In the case of $P^{\prime }=\left[1,0\right]$, P=[0,1], when the noise levels are 0,0.1,0.3 and 0.5, respectively, the average payoffs of reduction are 0.0230,0.0210,0.0188 and 0.0025, respectively. Figure 10 and Figure 11 also show that it is better to overestimate a dangerous enemy (i.e., the global-type attacker) than to underestimate it. This is because the global-type attacker who pays equal attention to the overall performance of the network as the defender will cause greater losses to the infrastructure network.

From Figure 2, we can find that with the increase of noise level α, the equilibrium payoff of the defender increases, whether in the face of global-type attacker or local-type attacker. The improvement of noise level α can effectively improve the final payoff of the defender. Meanwhile, by observing Figure 8 ($P^{\prime }=\left[0.5,0.5\right]$, P=[0.1,0.9]), Figure 9 ($P^{\prime }=\left[0.5,0.5\right]$, P=[0.9,0.1]), Figure 10 ($P^{\prime }=\left[1,0\right]$, P=[0,1]) and Figure 11 ($P^{\prime }=\left[1,0\right]$, P=[0,1]), we find that the final payoff of the defender is that the higher the noise level α is, the smaller the gap is between the false judgment of the prior probability of the two types of attackers and the correct judgment of the prior probability of the two types of attackers.

## 5. Conclusions and Discussions

With the emergence of terrorism, the protection of infrastructure has attracted the attention of more and more researchers. Moreover, with the continuous development of science and technology, infrastructure is becoming more interconnected, which makes infrastructure exhibit network characteristics. This allows us to consider infrastructure protection from a network perspective. The existing infrastructure network game model basically stays at the initial stage, assuming that both sides of attack and defense move at the same time, and both sides have complete information. For this reason, the Bayesian Stackelberg active deception defense game proposed in this paper mainly studies the defense actively transferring false network information to attackers under the condition of asymmetric information, and facing multiple types of attackers.

Firstly, we introduced a false network construction method to simulate the defender apply of asymmetric information to defend against the attacker actively.

Secondly, we apply the Bayesian Stackelberg game to simulate the reality that the defense of the infrastructure faces multiple types of attackers.

Finally, we conducted experiments on the scale-free network. Through experiments, we find that using false networks for active defense does improve the defender’s equilibrium return. After analyzing the attacker’s equilibrium strategy, it is found that the defender applies the ‘first-mover advantage’ to induce the attacker to adopt HAS whose attack success rate decreases. By analyzing the sensitivity of the prior probability of attacker type to the defender’s equilibrium payoff, we verify that the global-type attacker poses a greater threat to the infrastructure network. The defender should overestimate the probability of the dangerous attacker (i.e., a global-type attacker) rather than underestimate the probability of the dangerous attacker.

Although the model we built is closer to reality than that in previous research, we still have much room for improvement compared with the complexities of reality. Next, we need to consider irrational attackers based on our model. The existing research on using game theory to improve infrastructure network performance is based on rational attackers. However, in real life, an attacker is not necessarily rational. Modeling adversary bounded rational behavior should be considered. The SHARP (Stochastic Human behavior model with AttRactiveness and Probability weighting) model based on success or failure of the adversary’s past actions on exposed portions of the attack surface to model adversary adaptiveness [50] provides a good choice for our model of irrational attackers. Next, we will combine the bounded rational game model with network science to study the protection of infrastructure networks. Our research in this article is aimed at a single type of defender and multiple types of attackers, but the number of defenders and attackers is unique. In reality, multiple attackers and multiple defenders will also appear at the same time. Evolutionary game theory provides a train of thought for solving evolutionary stable strategy (ESS) by modeling a group dynamic game under the conditions of bounded rationality and incomplete information [51].

We note that the method of constructing a fake network is not unique, and its essence is to provide an incorrect or indeterminate degree of network nodes. The literature [52] gives us inspiration. Next, we will explore the influence of the information entropy of incomplete and imperfect information on offensive and defensive games from the perspective of information theory.
